# Clinical Safety-in-Use Study of a New Tampon Design

**DOI:** 10.1080/10647440300025504

**Published:** 2003

**Authors:** Stacey E. Shehin, Michaelle B. Jones, Anne E. Hochwalt, Frank C. Sarbaugh, Stephen Nunn

**Affiliations:** 1 Procter & Gamble Company 6110 Center Hill Avenue Cincinnati OH 45224 USA; 2 Arizona School of Health Sciences Phoenix AZ USA; 3 Hill Top Research Inc. Scottsdale AZ USA

## Abstract

*Objective:* To confirm the safety of a new experimental Tampax^®^ tampon and applicator compared with that of a currently marketed Tampax^®^ tampon and applicator using comprehensive gynecological and microbiological assessments.

*Methods:* A 2-month, single-blind, randomized, crossover study was conducted in which each subject served as her own control. Safety was evaluated by comparing potential product-related irritation (using colposcopic examination and subject diary data), assessment of vaginal discharge, vaginal pH, and effects on selected microorganisms (yeast, *Escherichia coli* ,*Staphylococcus aureus*  and group B streptococci) obtained by vaginal swab cultures after normal menstrual use in the experimental and control groups.

*Results:* In total, 110 women completed the study. There were no significant differences between the groups that used either the experimental or control tampon with regard to prevalence or mean cell density for the selected microorganisms. No differences were observed in the incidence or severity of erythema, in abrasion or ulceration of the cervix, vagina, introitus, vulva or perineum, or in mean vaginal pH and discharge assessments. There were equivalent low incidences of reported symptoms such as discomfort during insertion, wear or removal, and a similar low incidence of burning, stinging or itching during use of either the control or experimental tampon. There was a more favorable overall product rating for the experimental tampon (*p* = 0.003).

*Conclusions:* This approach provides a combination of gynecological, microbiological and self-reported (diary recall) methodologies in order to assess tampon safety during use more thoroughly than has previously been reported, and it supports a comparable safety profile for the experimental tampon and a currently marketed tampon.


*Infect Dis Obstet Gynecol 2003;11:89–99*

### Clinical safety-in-use study of a new tampon design

**Stacey E. Shehin[1], Michaelle B. Jones[1], Anne E. Hochwalt[1], Frank C. Sarbaugh[1] and Stephen Nunn[2,3]**

[1]*Procter & Gamble Company, Cincinnati, OH,*
[2]*Arizona School of Health Sciences, Phoenix, AZ and*
[3]*Hill Top Research, Inc., Scottsdale, AZ*

***Objective:*** To confirm the safety of a new experimental Tampax® tampon and applicator compared with that of a currently marketed Tampax® tampon and applicator using comprehensive gynecological and microbiological assessments.
***Methods:*** A 2-month, single-blind, randomized, crossover study was conducted in which each subject served as her own control. Safety was evaluated by comparing potential product-related irritation (using colposcopic examination and subject diary data), assessment of vaginal discharge, vaginal pH, and effects on selected microorganisms (yeast, *Escherichia coli*, *Staphylococcus aureus* and group B streptococci) obtained by vaginal swab cultures after normal menstrual use in the experimental and control groups.
***Results:*** In total, 110 women completed the study. There were no significant differences between the groups that used either the experimental or control tampon with regard to prevalence or mean cell density for the selected microorganisms. No differences were observed in the incidence or severity of erythema, in abrasion or ulceration of the cervix, vagina, introitus, vulva or perineum, or in mean vaginal pH and discharge assessments. There were equivalent low incidences of reported symptoms such as discomfort during insertion, wear or removal, and a similar low incidence of burning, stinging or itching during use of either the control or experimental tampon. There was a more favorable overall product rating for the experimental tampon ($p = 0.003$).
***Conclusions:*** This approach provides a combination of gynecological, microbiological and self-reported (diary recall) methodologies in order to assess tampon safety during use more thoroughly than has previously been reported, and it supports a comparable safety profile for the experimental tampon and a currently marketed tampon.

Key words: COLPOSCOPY; MICROFLORA; VAGINAL EPITHELIUM; TAMPONS

Tampons have been used for over 60 years as an internal method of absorbing menstrual flow. In the USA, tampons are regulated as a class-II medical device by the Food and Drug Administration (FDA), thus requiring FDA approval prior to marketing. Currently marketed tampons are composed of absorbents consisting of 100% cotton, 100% rayon, or blends of cotton and rayon. They are manufactured with or without an overwrap, and may be provided with a cardboard or plastic applicator for inserting the tampon into the vagina. Design and material changes to a currently marketed product that result in a significant change in function, or that may have a potential effect on safety, must be approved by the FDA. Therefore it is the responsibility of the tampon manufacturer to provide performance- and safety-related data to support the introduction of a

Correspondence to: Stacey E. Shehin, PhD, The Procter & Gamble Company, 6110 Center Hill Avenue, Cincinnati, OH 45224, USA. Email: shehin.se@pg.com

© 2003 The Parthenon Publishing Group new tampon to the market. A clinical safety-in-use study was conducted as part of a broader program which includes safety assessment of tampon raw materials, *in-vitro* toxic shock syndrome toxin-1 expression and vaginal microflora analyses[1].

The objective of this study was to compare a new tampon design with that of a tampon that has an established history of use by millions of women. Although clinical studies that assess the effects of tampon wear have been reported since 1942[2], there are no published studies that combine gynecological, microbiological and diary recall methodologies to assess in more detail safety-related end points associated with tampon use. The methods outlined in this study include evaluation of the condition of the lower genital tract tissues by colposcopy, and analysis of the prevalence of selected microorganisms. Previous experience in clinical studies indicates that a questionnaire completed by the subjects after product use effectively detects product-related sensations of burning, stinging and irritation (unpublished data). Questionnaires are widely used in medical surveillance and product evaluation, and have been found to be a valid and reliable method for subjective assessment of clinical symptoms[3–5].

### SUBJECTS AND METHODS

### Subjects

Healthy adult volunteers (aged 18–45 years) who normally used super-absorbency (9–12 g) tampons and had regular monthly menstrual cycles were recruited for the study and provided their signed informed consent. Potential subjects were evaluated by medical history, physical examination, and colposcopic and microbiology examinations for assessment of exclusion criteria. Specific exclusionary provisions included current or recent antibiotic or anti-inflammatory drug and/or steroid use, active vaginal or urogenital infection, and a history of herpes, toxic shock syndrome or any medical condition that might compromise immune-system functions. Subjects were eligible for entry to the study if they were deemed to be healthy by the principal investigator and if they met the inclusion criteria.

### Study products

Two Tampax® (Procter & Gamble Company, Cincinnati, OH) super-absorbency (9–12 g) tampon products were examined (Figure 1). The experimental product was a newly designed chevron-shaped pad with absorbent material knitted into the cord at the base of the pad. The experimental tampon components consisted of an absorbent pad made of cotton and rayon, a polyethylene and polypropylene overwrap, cotton stitching thread, cotton removal cord and a blue pearlescent plastic (polyethylene) applicator. The control product was a currently marketed super-absorbency Tampax® product consisting of a blended cotton and rayon fiber tampon with a non-woven rayon overwrap, cotton stitching thread and removal cord, and a flushable cardboard applicator. For women who required back-up menstrual protection, Always® Ultrathin Maxipads and Alldays® pantiliners (Procter & Gamble) were provided.

### Study design

This study had a randomized, controlled, crossover design and ran over two consecutive menstrual cycles. Subjects used the experimental tampon one month and the control product the other month, with the order of use (experimental/control or control/experimental) randomly assigned. The

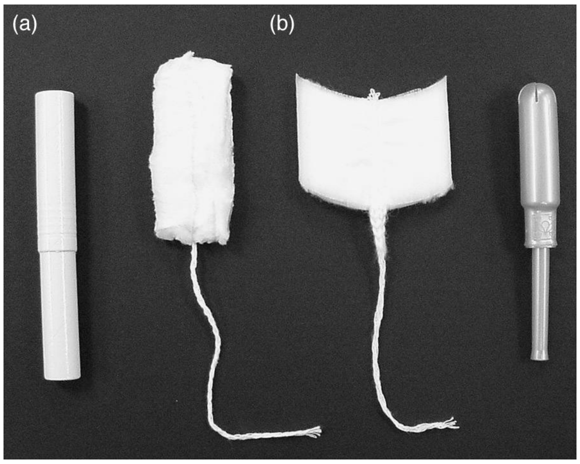

**Figure 1** Tampon products (prior to compression) used in the study. (a) Control tampon and applicator; (b) experimental tampon and applicator tampons were provided in plain coded wrappers. The number of products worn was left to the discretion of the subjects, who were instructed to insert, wear and withdraw the study tampons as they normally would, but not to wear two tampons at a time. The study design was approved by an Institutional Review Board prior to the beginning of the study.

The study subjects were also randomly assigned to one of two procedural groups, namely for microbiology or colposcopy. Study data were collected at visits 1 and 2, either during or following the subjects' menstrual cycles. Each subject kept a tampon-use diary and completed monthly comfort and product experience questionnaires. Table 1 summarizes the schedule of events.

### Examinations and laboratory tests

### *Microbiology assessments*

Samples were collected for microbiological analysis from the randomly assigned microbiology group subjects during day 3 or 4 of each of their menstrual cycles (visits 1 and 2). Vaginal swab samples were collected separately from the introitus and from the fornix near the cervix and were examined for selected microbial species (*Escherichia coli*, yeast species, *Staphylococcus aureus* and group B streptococci). *E. coli* was selected as an indicator of potential colonization from the lower gastrointestinal tract. Yeast species, *S. aureus* and group B streptococci represent endogenous flora of potential pathological significance[6].

Preweighed sterile Dacron swabs (Copan Italia, Brescia, Italy) were used for collection, with care taken to avoid contact with surfaces other than those designated for sampling. Samples were collected and then immediately plated, refrigerated and either analyzed on site or transported within 3 hours of collection to an additional laboratory for analysis. The media and methods used for isolation and identification were as follows.

Swabs were weighed before sampling and after sampling but prior to dilution and plating. After sampling, swabs were immediately placed in 5 ml of phosphate-buffered saline (PBS), mixed on a vortex for 10 seconds and plated on media as described below. The results were expressed as colony-forming units (CFUs) per gram of sample.

Colonies growing on mannitol salt agar (BBL 4321173) were identified by typical colonial morphology and Gram stain as staphylococci, with *S. aureus* identified by the catalase test and the Accu-Staph (Carr-Scarborough Microbiologicals, Inc., Decatur, GA) latex agglutination test for coagulase positive identification. Colonies of yeast growing on mycosel agar (BBL 4321847) were

**Table 1** Summary schedule of events

| *Procedure* | *Visit 0* *Pre-study* | *Visit 1* *Cycle 1* | *Visit 2* *Cycle 2 (Final)* |
|---|---|---|---|
| Consent form | X | | |
| Medical history questionnaire | X | | |
| Screening microbiological samples taken[a] | X | | |
| Screening colposcopic examination and vaginal assessment (all subjects) | X | | |
| Dispense tampons for menstrual use | X | X | |
| Tampon diary and monthly comfort questionnaire dispensed | X | X | |
| Dispense Always® products (if desired) | X | X | |
| Group 1 (microbiology sampling)[b] | | X | X |
| Group 2 (colposcopic examination) | | X | X |
| All subjects (vaginal assessment) | | X | X |
| Questionnaires completed | | X | X |

[a]Excluson criteria: diagnosis of bacterial vaginosis. *Trichomonas*, yeast infection, *Chlamydia* or *Neisseria gonorrhoeae*; [b]Samples taken for *Staphylococcus aureus*, group B streptococci, yeast and *E. coli*

identified by typical colony morphology and tested for germ-tube production. Germ-tube-positive organisms were identified as *Candida albicans*. Germ-tube-negative organisms (if present) were identified as 'yeast, not *C. albicans*,' and were speciated as *C. glabrata* or *C. tropicalis* using the API 20C (bioMérieux Vitek, Hazelwood, MO) test. Beta-hemolytic colonies growing on neomycin blood agar (BBL 4321791) were identified by typical colony morphology and Gram stain as streptococci, and group B streptococci were identified by the Christie, Atkins, Munch-Peterson (CAMP) test. *E. coli* growing on MacConkey II agar (BBL 4321172) was identified by typical colony morphology, Gram stain, indole production, citrate utilization and the API 20E (bioMérieux Vitek, Hazelwood, MO) test.

### *Colposcopy examination*

Colposcopic examination was performed on the randomly assigned colposcopy group subjects 24–48 hours after the use of the last tampon following each of their two study menstrual cycles (visits 1 and 2). The examination was performed in accordance with World Health Organization standards for colposcopic examination using a Leisegang Model 3-B colposcope (Berlin, Germany), and included visual examination of the perineum, vulva, introitus, vaginal walls, vaginal fornices and cervix[7]. For each location, the examiner noted the presence or absence of ulceration (defined as a tear in the epithelium, with a sharp area of demarcation), abrasion (defined as partially disrupted epithelium, with a diffuse area of demarcation) and/or erythema (defined as a vascular prominence noted on colposcopy, or obvious redness on gross inspection)[7].

Ulceration and abrasion were noted as being either present or absent, while erythema was rated on a four-point scale, where 0 = none, 1 = mild, 2 = moderate and 3 = severe.

### *Gynecological assessment*

Vaginal pH and vaginal discharge were assessed at the subjects' regular study visits 1 and 2. pH was measured using pH strips designed for measuring in the range 3.6–6.1 (JT Baker, Phillipsberg, NJ), and vaginal discharge was assessed as either normal or abnormal. If it was abnormal, vaginal discharge was assessed for color (white, gray, yellow, brown, red, green or other) and consistency (flocculent, menstruating, homogeneous, frothy, cheesy or other).

### *Diary and questionnaire assessments*

Information on the subjects' experience with the two study products was collected using a tampon use diary and a monthly comfort questionnaire, both of which were completed during or following each month of testing.

A tampon use diary sheet was completed for each study product used and was returned monthly following each period. Subjects were instructed to record the time of insertion and removal of each tampon, and to note any menstrual or vaginal symptoms (burning, stinging or itching sensation, cramping, tampon slipping down or out, or feeling that it was not properly in place). Subjects rated the product for overall comfort (very comfortable/comfortable/neither comfortable nor uncomfortable/uncomfortable/very uncomfortable) and noted any discomfort that was experienced while inserting, wearing or removing the tampon.

The monthly comfort questionnaire was completed at the end of each cycle. Subjects rated their study product overall (excellent/very good/good/fair/poor) and rated the comfort level (very comfortable/comfortable/neither comfortable nor uncomfortable/uncomfortable/very uncomfortable) of their study tampon. Space for additional comments was also provided.

### Statistical analyses

Summary statistics and 95% confidence intervals were generated for all parameters. All comparisons were conducted at the 0.05 significance level. To increase the power of detection of safety concerns relating to the test tampon, comparisons of certain parameters (e.g. microbial counts, erythema) were performed at the one-sided level. Comparisons of other parameters (e.g. vaginal pH and color assessments) were performed at the two-sided level.

For continuous parameters (e.g. microbial counts converted to $\log_{10}$ CFU/g and vaginal pH), experimental vs. control means were compared using the following two-period crossover analysis method.

For each subject, the treatment difference (experimental value minus control value) and visit difference (visit 2 value minus visit 1 value) were derived. The two-sample *t*-test, with treatment sequence as the factor, was performed on the treatment differences. If the result (a test for equal-period effect) was not statistically significant, then the null hypothesis was tested by applying the paired *t* test to the treatment differences. If the result for period effect was statistically significant, then the null hypothesis was tested by applying the two-sample *t*-test (with treatment sequence as the factor) to the visit differences. If parametric assumptions were not met, then the Wilcoxon rank-sum test and signed-rank test were performed.

For categorical data (e.g. microbiology data prevalence defined as the percentage of subjects with detectable CFU values, the percentage of subjects with erythema, the distributions of individual erythema scores, the percentage of subjects with positive abrasion and ulceration findings, the percentage of subjects with normal/abnormal vaginal discharge assessments and distributions of color/consistency), experimental versus control values were analyzed by means of stratified Cochran–Mantel–Haenszel tests.

### RESULTS

A total of 224 subjects were recruited and evaluated for inclusion and exclusion criteria. Of these, 53 subjects were dropped from the study before product use due to non-compliance with the protocol, or because they were ineligible according to the exclusion criteria. Thus a total of 171 subjects entered the product-use phase of the study. Of the 171 menstruating women who enrolled in the study and entered the product-use phase, 141 women completed the study (39 women in the microbiology group and 102 women in the colposcopy group). Of the 39 microbiology subjects who completed visit 2, five were not evaluable for the statistical analyses with microbial end points due to non-compliance with the protocol. Of the 102 colposcopy subjects who completed visit 2, 26 women were not evaluable for the statistical analyses of vaginal condition due to non-compliance with the protocol. No subjects dropped out because of a product-related adverse event.

The subjects had an average age of approximately 34 years (range 19–45 years). Most were Caucasian (94%), and the remainder were Hispanic or Asian. There were no statistically significant differences in tampon wear time between the control and experimental products (Table 2). The average wear time for subjects in the microbiology group was 5.1 hours for both control and experimental tampons. The average wear time for those in the colposcopy group was 5.4 and 5.5 hours for control and experimental tampons,

**Table 2** Average tampon weartime (WT) and daily number of tampons used per subject

| | *Experimental* | | | | *Control* | | | | |
|---|---|---|---|---|---|---|---|---|---|
| *Parameter* | *Mean* | *Minimum* | *Maximum* | *SE* | *Mean* | *Minimum* | *Maximum* | *SE* | *p-value* |
| *Microbiology group* (n = 35) | | | | | | | | | |
| Average WT (hours) | 5.1 | 2.5 | 9.7 | 0.25 | 5.1 | 2.1 | 8.4 | 0.27 | 0.905[a] |
| Average daily number | 3.6 | 1.0 | 10.0 | 0.26 | 3.8 | 1.5 | 9.3 | 0.24 | 0.212[a] |
| *Colposcopy group* (n = 97) | | | | | | | | | |
| Average WT (hours) | 5.5 | 2.2 | 10.7 | 0.16 | 5.4 | 2.7 | 9.6 | 0.14 | 0.245[b] |
| Average daily number | 3.2 | 1.3 | 8.0 | 0.10 | 3.1 | 1.7 | 7.5 | 0.09 | 0.265[a] |

[a]Two-sided *p*-value based on paired *t*-test; [b]Two-sided *p*-value based on two sample *t*-test on paired data; period effect detected;
SE, standard error respectively. Both control and experimental tampons were worn for ≤ 8.2 hours by the majority (95%) of subjects (data not shown).

### Microbiology results

Microbiology data were compiled and analyzed for 34 subjects who completed the study in compliance with the protocol. The prevalence of microorganisms detected at each sampling site (the cervix and introitus) for both products is summarized in Table 3. All yeasts present were identified as *C. albicans*. The prevalence of all assessed organisms was statistically similar ($p \geq 0.159$) for the control and experimental legs of the study (data not shown). Overall, the prevalence of the examined organisms was generally low, the highest prevalence being found for group B streptococci and *S. aureus* (up to 18% of subjects at either examined site). *E. coli* and *C. albicans* were identified in 9% and 6% of subjects at the cervix and introitus, respectively. The mean counts of examined organisms were similarly low, with mean $\log_{10}$ counts ranging from 0.1 to 1.1, and no statistically significant differences ($p \geq 0.50$) were found in the mean counts between the experimental and control legs at either the cervix or the introitus for any organism evaluated (data not shown).

### Colposcopy results

Colposcopy results were compiled and analyzed for 76 subjects who completed the study in compliance with the protocol. Table 4 summarizes the incidence of erythema, abrasion and ulceration at each individual examined anatomical site and the overall (i.e. at any examined site). It can be seen that the incidence of erythema was statistically similar between the experimental and control study legs ($p \geq 0.197$), and ranged from 41% to 99% and 36% to 100% during the experimental and control legs, respectively. The vast majority of erythema seen at any site was either mild or moderate (score = 1 or 2). Severe erythema was seen in only two subjects (2.6%) at two sites (cervical ectopy and vulva) in both the experimental and control legs, respectively. No severe erythema was seen in the other four anatomical sites that were examined (squamous mucosa, upper vagina, mid/low vagina and introitus) (data not shown).

Similarly, no statistically significant difference was found in the incidence of abrasion ($p \geq 0.841$) or ulceration ($p = 0.921$) between experimental and control product use. No incidence of abrasion or ulceration was seen at any site while the experimental product was being used. After use of the control product at visit 1, abrasion was observed in two subjects at each of two sites (middle/lower

**Table 3** Prevalence and cell density of microorganisms in positive cases

| | *Experimental* | | | *Control* | | |
|---|---|---|---|---|---|---|
| *Organism and site* | *Prevalence n (%)* | *Mean[a]* | *SE* | *Prevalence n (%)* | *Mean[a]* | *SE* |
| *Staphylococcus aureus* | | | | | | |
| Cervix | 3 (9) | 6.1 | 0.26 | 4 (12) | 4.5 | 0.39 |
| Introitus | 3 (9) | 6.1 | 0.53 | 6 (18) | 5.2 | 0.42 |
| *Yeast – Candida albicans* | | | | | | |
| Cervix | 1 (3) | 3.3 | 0.00 | 0 (0) | 0.0 | 0.00 |
| Introitus | 2 (6) | 4.4 | 0.15 | 2 (6) | 3.9 | 0.22 |
| *Group B streptococci* | | | | | | |
| Cervix | 6 (18) | 5.9 | 0.39 | 6 (18) | 5.8 | 0.35 |
| Introitus | 5 (15) | 6.3 | 0.35 | 6 (18) | 6.4 | 0.42 |
| *E. coli* | | | | | | |
| Cervix | 3 (9) | 4.6 | 0.75 | 3 (9) | 5.4 | 0.43 |
| Introitus | 4 (12) | 4.7 | 0.33 | 3 (9) | 5.0 | 0.38 |

[a]Mean cell densities expressed as $\log_{10}$ CFU/g. SE, standard error of the mean

**Table 4** Erythema, abrasion and ulceration incidence in colposcopy procedural group ($n = 76$)

| Type of incidence and genital site | Experimental | | Control | | |
|---|---|---|---|---|---|
| | $n^a$ | $\%^a$ | $n^a$ | $\%^a$ | $p$-value[b] |
| *Erythema* (> 0)[c] | | | | | |
| Any site | 75 | 99 | 76 | 100 | 0.841 |
| Cervix | | | | | |
| Squamous mucosa | 71 | 94 | 71 | 93 | 0.500 |
| Ectopy | 31 | 41 | 27 | 36 | 0.197 |
| Upper vagina | 58 | 77 | 63 | 83 | 0.841 |
| Mid/low vagina | 64 | 84 | 65 | 86 | 0.591 |
| Introitus | 66 | 87 | 72 | 95 | 0.958 |
| Vulva | 68 | 90 | 74 | 97 | 0.983 |
| *Presence of abrasion* | | | | | |
| Any site | 0 | 0 | 2 | 3 | 0.921 |
| Cervix | | | | | |
| Squamous mucosa | 0 | 0 | 0 | 0 | — |
| Ectopy | 0 | 0 | 0 | 0 | — |
| Upper vagina | 0 | 0 | 2 | 3 | 0.921 |
| Mid/low vagina | 0 | 0 | 1 | 1 | 0.841 |
| Introitus | 0 | 0 | 0 | 0 | — |
| Vulva | 0 | 0 | 0 | 0 | — |
| *Presence of ulceration* | | | | | |
| Any site | 0 | 0 | 2 | 3 | 0.921 |
| Cervix | | | | | |
| Squamous mucosa | 0 | 0 | 0 | 0 | — |
| Ectopy | 0 | 0 | 0 | 0 | — |
| Upper vagina | 0 | 0 | 2 | 3 | 0.921 |
| Mid/low vagina | 0 | 0 | 2 | 3 | 0.921 |
| Introitus | 0 | 0 | 0 | 0 | — |
| Vulva | 0 | 0 | 0 | 0 | — |

[a] *n* and % = number and percentage, respectively, of subjects with incidence at genital sampling site; [b] one-sided *p*-values based on stratified Cochrane–Mantel–Haenszel test; [c] data for subjects with any erythema (i.e. erythema score > 0, where 0 = none, 1 = mild, 2 = moderate and 3 = severe)

vagina and upper vagina), and the same two subjects had ulcerations at the same two sites (incidence ≤ 3%). No medical follow-up was necessary in either of these cases. At visit 2, the results of the colposcopic examination showed no abrasion or ulceration in these subjects.

### Gynecological results

Vaginal pH and discharge data were analyzed for a smaller number of subjects than the other study parameters, due to lack of usable data. In total, vaginal pH was analyzed for 10 microbiology group subjects and 60 colposcopy group subjects, and the results are summarized in Table 5. It can be seen that the mean pH values were very consistent between the experimental and control legs in each procedural group, with mean experimental versus control values of 5.4 versus 5.1 and 4.8 versus 4.7 for the microbiology and colposcopy groups, respectively. No statistically significant differences were found between the two study legs in either procedural group (microbiology, $p = 0.421$; colposcopy, $p = 0.857$).

Most vaginal discharge was assessed as normal for both experimental and control products, namely 97 versus 81% for the 32 microbiology subjects ($p = 0.971$) and 96 versus 95% for the 75

**Table 5** Gynecological assessment summary for microbiology and colposcopy groups

| *Parameter* | *Experimental* | *Control* | *p-value* |
|---|---|---|---|
| *Vaginal pH* | | | |
| Microbiology group ($n$ = 10)[a] | | | |
| Mean (SE) | 5.4 (0.24) | 5.1 (0.19) | 0.421[c] |
| Colposcopy group ($n$ = 60)[a] | | | |
| Mean (SE) | 4.8 (0.10) | 4.7 (0.09) | 0.857[c] |
| *Vaginal discharge* | | | |
| Microbiology group ($n$ = 32)[a] | | | |
| Normal: $n$ (%)[b] | 31 (97%) | 26 (81%) | |
| Abnormal: $n$ (%)[b] | 1 (3%) | 6 (19%) | 0.971[d] |
| Colposcopy group ($n$ = 75)[a] | | | |
| Normal: $n$ (%)[b] | 72 (96%) | 71 (95%) | |
| Abnormal: $n$ (%)[b] | 3 (4%) | 4 (5%) | 0.673[d] |
| *Vaginal discharge consistency* | | | |
| Colposcopy group ($n$ = 75)[a] | | | |
| Menstruating: $n$ (%)[b] | 2 (3%) | 4 (5%) | 0.549[e] |
| Homogeneous: $n$ (%)[b] | 1 (1%) | 0 (0%) | |

[a]$n$ = number of evaluable subjects for whom experimental and control data are not missing; [b]$n$ = number of subjects with category of response; [c]Two-sided $p$-value for vaginal pH based on paired $t$-test; [d]One-sided $p$-value for abnormal vaginal discharge based on stratified Cochrane–Mantel–Haenszel (CMH) test; [e]Two-sided $p$-value for consistency based on stratified CMH general association test; SE, standard error of the mean colposcopy subjects ($p = 0.673$), respectively. For those microbiology subjects who were rated as 'non-normal,' the only color noted was 'red' and the only consistency noted was 'menstruating' (microbiology subjects were examined during their menstrual periods). Among the colposcopy subjects with discharge who were rated as 'non-normal,' the consistency assessment was either 'menstruating' or 'homogenous,' and was not significantly different between the experimental and control legs ($p = 0.549$). No color assessments were noted for colposcopy group cases with non-normal vaginal discharge.

### Diary and questionnaire results

Product acceptance was assessed using a monthly product questionnaire and tampon use diary. The questionnaire and diary data indicated consistently positive assessments of both study tampons, although there was a more favorable overall product rating for the experimental tampon ($p = 0.003$) (data not shown). When assessing the experimental tampon, 92% of colposcopy subjects and 91% of microbiology subjects rated it as good to excellent overall, with positive to neutral comfort ratings given by 94% of the colposcopy subjects and 100% of the microbiology subjects. These results are similar to the 88 and 89% of good to excellent overall ratings and 94 and 95% of positive to neutral comfort ratings given to the control tampon in the colposcopy and microbiology groups, respectively. There were equivalent ($p \geq 0.982$) low incidences of reported menstrual-related symptoms such as discomfort during insertion, wear or removal ($\leq 12.4\%$), and vaginal symptoms such as burning, stinging or itching ($\leq 1.7\%$).

### DISCUSSION

Menstrual tampons have been widely marketed for over 60 years and are currently used by up to 50% of menstruating women in industrialized countries[8]. In addition to this long history of use and acceptance, the safety of tampons has been documented over the years in clinical studies[9–11]. Although the parameters examined in these studies have varied, they have generally included assessment of potential changes in the microflora of the vagina and/or possible mechanical irritation effects associated with tampon use. This report is the first of its kind to combine gynecologic, microbiological and diary assessment methods to evaluate the safety of tampon use.

More recently, as new tampons have been developed, studies have been conducted to compare the safety profiles of various tampons, with comparisons being made between tampons made of different absorbent materials and/or tampons with different degrees of absorbency[8,12–15]. This was the central purpose of this study, namely to compare the safety of a currently marketed tampon (Tampax® super-absorbency tampon) with a newly designed tampon with the same absorbency capability. As part of an ongoing research and product development effort, the present study was designed both to ascertain the safety-in-use profile of the new tampon and to contribute to the body of knowledge and understanding of vaginal health and tampon use.

Our primary goal was to compare a new tampon shape, absorbent braid and plastic applicator with those of a tampon with an established history of use. To achieve this, we designed a single-blind, crossover, clinical safety-in-use study in which each woman served as her own control. In order to ensure a realistic scenario, subjects were required to be current users of super-absorbency tampons and were instructed to use the study products as they would use their normal menstrual protection. Microbiological and colposcopic examinations were performed, with each subject assigned to either the microbiological or colposcopic group. In addition, two gynecological examinations per subject (including measurement of vaginal pH and assessment of vaginal discharge) were performed. Microbiological parameters included the prevalence and mean counts of four species (*S. aureus*, *C. albicans*, group B streptococci and *E. coli*), and colposcopy examination included assessment of erythema, abrasion and ulceration at six anatomical sites (perineum, vulva, introitus, vaginal walls, vaginal fornices and cervix).

The average tampon wear time in this study (5.1–5.5 hours) is consistent with the results obtained from consumer test studies using the same tampons in which the average tampon wear time was 5.2 hours for both control and experimental products (unpublished data).

The crossover comparisons of all microbial parameters in this study indicated that there were no statistically significant differences between the experimental and control tampon. The microbiology assessments of 34 subjects indicated a similar prevalence of the four individual species assessed. Similarly, mean $\log_{10}$ counts were also consistent between the two study arms. As such, these findings with regard to comparison of the effect of two different designs of tampons using similar fibers are consistent with previous studies which have found that tampon use has little effect on the vaginal microflora[9,12–15].

The prevalence figures of *S. aureus*, *E. coli* and Group B streptococci in positive cases (Table 3) were similar to the values found in the published literature on tampon users[9,16–19], but the prevalence of *C. albicans* in this study (3–6%) is lower than previously reported values (11–19%)[9,17].

Crossover comparison of visual parameters as assessed by the colposcopic examination of 76 subjects in this study also revealed no statistically significant differences in the incidence of lower genital tract irritation between the two tampons, as recorded by erythema and subjective data. Although mild erythema was frequently present, abrasion or ulceration were rare, and were seen in only two of the subjects. As in the published literature, our results showed that erythema or other minor vaginal irritation is not uncommon, and in fact has also been shown to occur in women who use external sanitary protection[20]. The rare incidence of more serious effects, such as ulceration, is similarly consistent with other studies of normal menstrual use of tampons which found ulceration as an uncommon type of lesion[20–22]. There have been few studies that have compared product-related irritation between different tampons[8], as most of the published literature has dealt with ulceration associated with non-typical tampon use, including daily use of super-absorbent products for many months[23,24]. However, our results confirm those of studies of tampons in normal menstrual use, which have generally found no significant differences in irritation between different tampon fibers or absorbency levels[15,20,21].

There were no differences in vaginal pH between users of the experimental and control tampon in either the microbiology or colposcopy group. The higher mean pH for the microbiology group compared with the colposcopy group is probably due to the different days during the menstrual cycle when the measurements were obtained. The microbiology group measurements were made on either day 3 or day 4 of each menstrual period, and the colposcopy group measurements were made 1 to 2 days after each menstrual period. The pH of the vagina is higher (i.e. less acidic) during menstruation, probably due to the alkalinity of the blood that is present[25], and our results are consistent with previously reported values of vaginal pH during and after menstruation[26].

The results of the tampon use diaries and monthly questionnaires that were used to assess additional safety-related end points such as burning, stinging or itching revealed no subjective evidence of vaginal effects due to use of either the control or experimental tampon.

In conclusion, the data obtained from this crossover study demonstrate that normal menstrual use of the new chevron-shaped tampon with absorbent braid and plastic applicator is unlikely to have an adverse effect on vaginal health. The colposcopic, microbiological and diary records that were used to evaluate objectively and subjectively vaginal condition during tampon use provide a sensitive and thorough method for comparing the safety of different tampon designs.

### ACKNOWLEDGMENTS

The authors would like to thank Ronald W. Berg and Tommie L. James for their expert microbiological assistance.

### REFERENCES

1. US Food and Drug Administration, Center for Devices and Radiological Health. *Draft Guidance for the Content of Premarket Notifications for Menstrual Tampons*. US Food and Drug Administration, Center for Devices and Radiological Health, Rockville, MD, 1995:1–10
2. Magid MO, Geiger J. The intravaginal tampon in menstrual hygiene: a clinical study. *Med Record* 1942;155:316
3. Robinson MK, Perkins MA. Evaluation of a quantitative clinical method for assessment of sensory skin irritation. *Contact Dermatitis* 2001;45:205–13
4. Kjaergaar SK, Hodgson M. The assessment of irritation using clinical methods and questionnaires. *Am Ind Hyg Assoc J* 2001;62:711–16
5. Shaw C, Matthews RJ, Perry SI, *et al*. Validity and reliability of an interviewer-administered questionnaire to measure the severity of lower urinary tract symptoms of storage abnormality: the Leicester Urinary Symptom Questionnaire. *Br J Urol* 2002;90:205–15
6. Hochwalt AE, Berg RW, Meyer SJ, *et al*. Site-specific prevalence and cell densities of selected microbes in the lower reproductive tract of menstruating tampon users. *Infect Dis Obstet Gynecol* 2002;10:1–11
7. World Health Organization. *Manual for the Standardization of Colposcopy for the Evaluation of Vaginally Administered Products*. Geneva: World Health Organization, 1996:5–8
8. Raudrant D, Landrivon G, Frappart L, *et al*. Comparison of the effects of different menstrual tampons on the vaginal epithelium: a randomized clinical trial. *Eur J Obstet Gynecol Reprod Biol* 1995; 58:41–6
9. Morris CA, Morris DF. 'Normal' vaginal microbiology of women of childbearing age in relation to the use of oral contraceptives and vaginal tampons. *J Clin Pathol* 1967;20:636–40
10. Smith CB, Noble V, Bensch R, *et al*. Bacterial flora of the vagina during the menstrual cycle. *Ann Intern Med* 1982;96:948–51
11. Onderdonk AB, Zamarchi GR, Walsh JA, *et al*. Methods for quantitative and qualitative evaluation of vaginal microflora during menstruation. *Appl Environ Microbiol* 1986;51:333–9
12. Onderdonk AB, Zamarchi GR, Rodriguez ML, *et al*. Quantitative assessment of vaginal microflora during use of tampons of various compositions. *Appl Environ Microbiol* 1987;53:2774–8
13. Onderdonk AB, Zamarchi GR, Rodriguez ML, *et al*. Qualitative assessment of vaginal microflora during use of tampons of various compositions. *Appl Environ Microbiol* 1987;53:2779–84
14. Onderdonk AB, Delaney ML, Zamarchi GR, *et al*. Normal vaginal microflora during use of various forms of catamenial protection. *Rev Infect Dis* 1989;11(Suppl. 1):S61–7
15. Hochwalt AE, Jones MA, Sarbaugh FC, *et al.* Clinical safety in use of a layered-fiber tampon. *Obstet Gynecol* 2001;97(Suppl. 1):S19–20
16. Chow AW, Percival-Smith R, Bartlerr KH, *et al.* Vaginal colonization with *Escherichia coli* in healthy women: determination of relative risks by quantitative culture and multivariate statistical analysis. *Am J Obstet Gynecol* 1986;154:120–6
17. Chow AW, Bartlett KH, Percival-Smith R, *et al.* Vaginal colonization with *Staphylococcus aureus*, positive for toxic-shock marker protein, and *Escherichia coli* in healthy women. *J Infect Dis* 1984;150:80–4
18. Martin RR, Buttram V, Besch P, *et al.* Nasal and vaginal *Staphylococcus aureus* in young women: quantitative studies. *Ann Intern Med* 1982;96:951–3
19. Persson K, Bjerre B, Hansson H, *et al.* Several factors influencing the colonization of group B streptococci – rectum probably the main reservoir. *Scand J Infect Dis* 1981;13:171–5
20. Berkeley AS, Micha JP, Freedman KS, *et al.* The potential of digitally inserted tampons to induce vaginal lesions. *Obstet Gynecol* 1985;66:31–5
21. Friedrich EG, Siegesmund KA. Tampon-associated vaginal ulcerations. *Obstet Gynecol* 1980; 55:149–56
22. Fraser IS, Lähteenmäki P, Elomaa K, *et al.* Variations in vaginal epithelial surface appearance determined by colposcopic inspection in healthy, sexually active women. *Hum Reprod* 1999; 14:1974–8
23. Barrett KR, Bledsoe S, Greer BE, *et al.* Tampon-induced vaginal or cervical ulceration. *Am J Obstet Gynecol* 1977;127:332–3
24. Danielson RW. Vaginal ulcers caused by tampons. *Am J Obstet Gynecol* 1983;146:547–8
25. Rakoff AE, Feo LG, Goldstein L. The biologic characteristics of the normal vagina. *Am J Obstet Gynecol* 1944;47:467–94
26. Wagner G, Ottesen B. Vaginal physiology during menstruation. *Ann Intern Med* 1982;96:921–3

Received 09/23/02; Accepted 04/22/03

## References

[PDF_01144] Barrett K. F., Bledsoe S., Greer B. E., Droegemueller W. (1977). Tampon-induced vaginal or cervical ulceration.. Am J Obstet Gynecol.

[PDF_01133] Berkeley A. S., Micha J. P., Freedman K. S., Hirsch J. C. (1985). The potential of digitally inserted tampons to induce vaginal lesions.. Obstet Gynecol.

[PDF_01121] Chow A. W., Bartlett K. H., Percival-Smith R., Morrison B. J. (1984). Vaginal colonization with Staphylococcus aureus, positive for toxic-shock marker protein, and Escherichia coli in healthy women.. J Infect Dis.

[PDF_01116] Chow A. W., Percival-Smith R., Bartlett K. H., Goldring A. M., Morrison B. J. (1986). Vaginal colonization with Escherichia coli in healthy women. Determination of relative risks by quantitative culture and multivariate statistical analysis.. Am J Obstet Gynecol.

[PDF_01147] Danielson R. W. (1983). Vaginal ulcers caused by tampons.. Am J Obstet Gynecol.

[PDF_01139] Fraser I. S., Lähteenmäki P., Elomaa K., Lacarra M., Mishell D. R., Alvarez F., Brache V., Weisberg E., Hickey M., Vallentine P. (1999). Variations in vaginal epithelial surface appearance determined by colposcopic inspection in healthy, sexually active women.. Hum Reprod.

[PDF_01136] Friedrich E. G., Siegesmund K. A. (1980). Tampon-associated vaginal ulcerations.. Obstet Gynecol.

[PDF_01060] Kjaergaar S. K., Hodgson M. (2001). The assessment of irritation using clinical methods and questionnaires.. AIHAJ.

[PDF_01126] Martin R. R., Buttram V., Besch P., Kirkland J. J., Petty G. P. (1982). Nasal and vaginal Staphylococcus aureus in young women: quantitative studies.. Ann Intern Med.

[PDF_01083] Morris C. A., Morris D. F. (1967). Normal vaginal microbiology of women of childbearing age in relation to the use of oral contraceptives and vaginal tampons.. J Clin Pathol.

[PDF_01098] Onderdonk A. B., Zamarchi G. R., Rodriguez M. L., Hirsch M. L., Muñoz A., Kass E. H. (1987). Qualitative assessment of vaginal microflora during use of tampons of various compositions.. Appl Environ Microbiol.

[PDF_01094] Onderdonk A. B., Zamarchi G. R., Rodriguez M. L., Hirsch M. L., Muñoz A., Kass E. H. (1987). Quantitative assessment of vaginal microflora during use of tampons of various compositions.. Appl Environ Microbiol.

[PDF_01090] Onderdonk A. B., Zamarchi G. R., Walsh J. A., Mellor R. D., Muñoz A., Kass E. H. (1986). Methods for quantitative and qualitative evaluation of vaginal microflora during menstruation.. Appl Environ Microbiol.

[PDF_01129] Persson K., Bjerre B., Hansson H., Forsgren A. (1981). Several factors influencing the colonization of group B streptococci--rectum probably the main reservoir.. Scand J Infect Dis.

[PDF_01078] Raudrant D., Landrivon G., Frappart L., De Haas P., Champion F., Ecochard R. (1995). Comparison of the effects of different menstrual tampons on the vaginal epithelium: a randomised clinical trial.. Eur J Obstet Gynecol Reprod Biol.

[PDF_01056] Robinson M. K., Perkins M. A. (2001). Evaluation of a quantitative clinical method for assessment of sensory skin irritation.. Contact Dermatitis.

[PDF_01063] Shaw C., Matthews R. J., Perry S. I., Assassa R. P., Williams K., McGrother C., Dallosso H., Jagger C., Mayne C., Clarke M. (2002). Validity and reliability of an interviewer-administered questionnaire to measure the severity of lower urinary tract symptoms of storage abnormality: the Leicester Urinary Symptom Questionnaire.. BJU Int.

[PDF_01087] Smith C. B., Noble V., Bensch R., Ahlin P. A., Jacobson J. A., Latham R. H. (1982). Bacterial flora of the vagina during the menstrual cycle: findings in users of tampons, napkins, and sea sponges.. Ann Intern Med.

[PDF_01152] Wagner G., Ottesen B. (1982). Vaginal physiology during menstruation.. Ann Intern Med.

